# Analysis of the candidate 8p21 tumour suppressor, *BNIP3L*, in breast and ovarian cancer

**DOI:** 10.1038/sj.bjc.6600674

**Published:** 2003-01-28

**Authors:** J Lai, J Flanagan, W A Phillips, G Chenevix-Trench, J Arnold

**Affiliations:** 1 The Queensland Institute of Medical Research, PO Box Royal Brisbane Hospital, Herston, Queensland 4006, Australia; 2 Peter MacCallum Cancer Institute, St Andrews' Place, East Melbourne, Victoria 3002, Australia; 3 Department of Pathology, University of Queensland, St Lucia, Brisbane, Queensland 6067, Australia

**Keywords:** BNIP3L, DHPLC, 8p21, LOH, cancer

## Abstract

Loss of heterozygosity (LOH) on the short arm of chromosome 8, at 8p12–p23, is one of the most frequent genetic events in both breast and ovarian cancer, suggesting the location of a shared tumour suppressor gene. Microcell-mediated chromosome transfer of chromosome 8 suppresses tumorigenicity and growth of colorectal and prostate cancer cell lines, further supporting the presence of a tumour suppressor gene on 8p. We have taken a candidate gene approach to try to identify this tumour suppressor gene at 8p12–p23. *BNIP3L*, which has sequence homology to pro-apoptotic proteins and the ability to suppress colony formation in soft agar, is located at 8p21, within a region of ovarian cancer LOH, breast cancer LOH and prostate cancer metastasis suppression. *BNIP3L* expression was assessed by both RT–PCR and Northern blot analysis in breast and ovarian cancer cell lines and found to be expressed at similar levels relative to expression in their respective normal epithelial cell lines. Genetic analysis of *BNIP3L* in 40 primary ovarian and 25 primary breast tumours identified one somatic, intronic mutation in one ovarian tumour, as well as several polymorphisms, including one resulting in an amino-acid substitution. These data suggest that *BNIP3L* is unlikely to be the target of 8p LOH in ovarian or breast cancer.

Breast and ovarian cancers are the leading and fifth leading causes of cancer deaths, respectively, in women in developed countries. Both cancers are epithelial in origin and share some common genetic and epidemiological risk factors (Claus *et al*, 1996). While some of the genes involved in predisposition to familial breast and ovarian cancer are known (Claus *et al*, 1996; Welsch and King, 2001), little is understood about the somatic molecular changes that occur during the development of sporadic breast and ovarian cancer. Few genes have been found to be frequently down-regulated in these tumours, and even fewer to carry frequent mutations (Liu and Ganesan, 2002).

The highest rates of loss of heterozygosity (LOH) in breast cancer are found at 17p, 8p and 7q, while in ovarian cancer they are at 17p, 17q, 8p, 22q and 18q (Callahan *et al*, 1992; Shelling *et al*, 1995; Phelan *et al*, 1998; Seitz *et al*, 2000; Pribill *et al*, 2001). LOH has been reported at 8p12–8p23 in over 50% of primary breast tumours (Wang *et al*, 1999; Yokota *et al*, 1999). Similarly, LOH occurs in over 50% of ovarian tumors at p12–p23 (Brown *et al*, 1999; Wright *et al*, 1998). The occurrence of this common region of LOH in breast and ovarian cancer suggests that 8p12–23 probably harbours one or more tumour suppressor genes that are somati-cally inactivated in these neoplasms.

The *BNIP3L* gene is located between markers D8S1752 and D8S1989 that map to 8p21 in the smallest region of overlap (SRO) identified by LOH analysis of breast and ovarian tumours (Seitz *et al*, 2000; Brown *et al*, 1999), and to a region of prostate cancer metastasis suppression (Nihei *et al*,, 1996*).* *BNIP3L* encodes a protein that is homologous to the proapoptotic protein BNIP3 (Matsushima *et al*, 1998; Chen *et al*, 1999; Imazu *et al*, 1999; Yasuda *et al*, 1999). Several well-characterised tumour suppressor genes such as *PTEN*, *p53* and *RB* have been shown to function through the control of apoptosis (Bellamy, 1997; Zornig *et al*, 2001), making *BNIP3L* a good candidate for an 8p tumour suppressor gene. Like its homologue, *BNIP3L* contains a sequence motif for a transmembrane domain and a putative proapoptotic BH3 (Bcl-2 homology-3) domain (Yasuda *et al*, 1999). *BNIP3L* interacts with the antiapoptotic viral proteins E1B19kD and BCL2 to induce cell death by altering mitochondrial membrane permeability (Chen *et al*, 1999; Imazu *et al*, 1999; Yasuda *et al*, 1999). Further evidence for a possible tumour suppressor role of the *BNIP3L* gene is provided by experiments showing that the clonicity in soft agar of cervical cancer cell lines was suppressed after transfection of the *BNIP3L* gene (Matsushima *et al*, 1998). Considering its function and location, *BNIP3L* is a strong candidate for the breast and/or ovarian cancer tumour suppressor gene located at 8p21.

In order to determine whether *BNIP3L* functions as a tumour suppressor gene in breast and/or ovarian cancer, we have analysed its expression in ovarian and breast cancer cell lines and screened all exons for mutations in a panel of primary ovarian and breast cancers.

## Materials and Methods

### Cell lines

Human ovarian surface epithelial cell lines (HOSE) 1.1 and 17.1, immortalised with a retroviral vector expressing human papillomavirus oncogenes (Tsao *et al*, 1995), were cultured in RPMI 1640 with 10% FCS. The human ovarian cancer cell lines 27 27/87, A2780, CAOV3, CI8O135, COLO316, JAM, OAW 42, PEO1, PEO14, SKOV3 and OVCAR 3 were maintained in RPMI 1640 with 10% FCS, and HEY, DOV-13, OVCA 432, OVCAR-4, OVCAR-5 and OVCAR-8 were maintained in MEM alpha with 10% FCS. OVCA 420 was maintained in 1 : 1 MCDB105 : M199 with 10% FCS and 59M was maintained in DMEM with 10% FCS.

The human breast epithelial cell lines Bre-80-hTERT1 and Bre-80-hTERT2 (gifts of R Reddel), immortalised with human telomerase cDNA, were cultured in 1 : 1 RPMI 1640 : MCDB 170 with 10% FCS, and MCDB 170 with 10% FCS respectively. The human breast cancer cell lines BC 312, NB88, T-47D, ZR-75-1, BT-20, MDA-MB-231, BT474 and BT483 were maintained in RPMI 1640 with 10% FCS with the latter two supplemented with 1 *μ*g ml^−1^ insulin. The cell lines 21MT-1, 21MT-2 and 21NT were maintained in MEM alpha with 10% FCS, 10 mM HEPES, 1 *μ*g ml^−1^ insulin, 2.8 *μ*M hydrocortisone and 0.1 mM nonessential amino acids. SK-BR-3 was maintained in DMEM with 10% FCS and MCF7 was maintained in MEM alpha with 10% FCS and 1 *μ*g ml^−1^ insulin.

### Primary tumours

Primary ovarian tumour cells were obtained from 40 patients with malignant ovarian neoplasms undergoing surgery (Table 2

**Table 2:** LOH status, mutation results and clinicopathological features of primary ovarian tumours

	**Clinicopathology**						**LOH**			**Mutations or SNPs**				
**Case**	**Histo**	**Stage**	**Grade**	**D8S137**	**D8S1048**	**Spanning exon**	**nt^a^**	**Codon**	**Base change**	**a.a change**				
35/87	SER	3C	3	LOH	LOH	5	25044	137	CTT→TTT^b^	Leu→Phe
69/89	SER	3	2	NI	LOH					
35/90	SER	3B	2/3	NL	NL	4	24147	IVS3-56	Del G^b^ and T→C^a^	Intronic
5/91	SER	2A	2/3	NI	NL		24752	IVS3-61		Intronic
23/91	ENDO	3	2	LOH	LOH	5	25199	IVS5+15	A→G	Intronic
33/91	ENDO	4	3	LOH	LOH					
44/91	SER	3C	3	NI	LOH	5	25199	IVS5+15	A→G	Intronic
45/91	SER	3C	NS	LOH	LOH					
52/91	SER	2B	2	NI	NL	5	25199	IVS5+15	A→G	Intronic
59/91	SER	3	3	NL	NI	5	25199	IVS5+15	A→G	Intronic
68/91	SER	3	3	NL	NL					
2/92	SER	3	2	NL	NI	5	25199	IVS5+15	A→G	Intronic
3/92	SER	3	3	LOH	LOH	5	25199	IVS5+15	A→G	Intronic
4/92	SER	3B	3	NI	NL					
23/92	SER	3	3	CR	CR					
26/92	CCC	4	2	LOH	LOH	2	8194	61	TCA→TCT^b^	Ser→Ser
						5	25199	IVS5+15	A→G	Intronic
39/92	SER	3	2	NI	NI					
52/92	SER	3	2	NI	NL	5	25199	IVS5+15	A→G	Intronic
62/92	SER	3C	2/3	LOH	LOH					
66/92	ENDO	3C	3	NI	NL					
67/92	ENDO	1C	2/3	NL	NL					
76/92	SER	3	2/3	LOH	NI	5	25199	IVS5+15	A→G	Intronic
3/93	SER	3C	3	NL	CR	2	8243	IVS2+11	C→T	Intronic
4/93	SER	3	1/2	NL	CR	5	25199	IVS5+15	A→G	Intronic
5/93	SER	3C	2/3	NI	NI					
6/93	SER	3C	2/3	LOH	NI					
8/93	SER	3C	2/3	LOH	LOH					
10/93	SER	3C	2/3	NL	NL	5	25199	IVS5+15	A→G	Intronic
12/93	CCC	3	2/3	NI	NL	2	8209	66	TCC→TCA^b^	Ser→Ser
13/93	SER	3C	NS	NI	NI					
35/93	SER	3C	3	NI	LOH					
61/93	ENDO	4	2	NI	LOH	2	8209	66	TCC→TCG^b^	Ser→Ser
						5	25199	IVS5+15	A→G	Intronic
65/93	ENDO	3	2/3	NI	NI	5	25199	IVS5+15	A→G	Intronic
99/93	ENDO	4	3	NL	NL	5	25199	IVS5+15	A→G	Intronic
108/93	SER	3C	2	NL	NI	5	25199	IVS5+15	A→G	Intronic
14/94	SER	1A	NS	NL	NL					
23/94	SER	3	3	NI	NL	5	25199	IVS5+15	A→G	Intronic
25/94	SER	3C	2	NL	NL					
34/94	SER	3C	3	NI	ND	5	25199	IVS5+15	A→G	Intronic
70/94	SER	3C	2	NL	ND					

a Bases counted from ATG start codon; NS=not specified; NL=no loss; LOH=loss of heterozygosity; NI=not informative; CR=can't read; ND=not done.

). There were 31 serous tumours, seven endometrioid tumours and two clear cell tumours. All patients were staged at laparotomy, in accordance with the recommendations of the International Federation of Gynaecology and Obstetrics (FIGO). Primary breast tumours were obtained from 25 patients with malignant tumours undergoing surgery (Table 3

**Table 3:** LOH status, mutation results and clinicopathological features of primary breast tumours

	**Clinicopathology**						**LOH**			**Mutations or SNPs**				
**Case**	**Histo**	**Stage**	**Grade**	**D8S137**	**D8S1048**	**Spanning exon**	**nt^a^**	**Codon**	**Base change**	**a.a change**				
1	Ductal	IIIA	1	LOH	NI	5	25199	IVS5+15	A→G	Intronic
2	Lobular	IIIA	2	NL	CR					
3	NS	IIB	3	CR	LOH					
4	Med/duct	IIB	3	NI	CR	5	25199	IVS5+15	A→G	Intronic
5	Ductal	IIIB	2	NI	NI					
6	Ductal	IIIB	2	NI	NI					
7	Ductal	NS	2	NI	NL	5	25199	IVS5+15	A→G	Intronic
8	Ductal	IIB	NS	LOH	NI					
9	Ductal	IIA	2	LOH	NI					
10	Ductal	IIB	3	NL	NL	5	25199	IVS5+15	A→G	Intronic
11	NS	IIB	1	NL	NL	5	25199	IVS5+15	A→G	Intronic
12	Lob/duct	NS	2	NI	NI					
13	Ductal	IIIA	3	LOH	NL	5	25199	IVS5+15	A→G	Intronic
14	Ductal	IIA	2	NL	NL					
15	Ductal	IIB	3	NL	NL	5	25199	IVS5+15	A→G	Intronic
16	Ductal	IIA	2	NI	NI	5	25199	IVS5+15	A→G	Intronic
17	Ductal	IIA	3	NL	NL	5	25199	IVS5+15	A→G	Intronic
18	Ductal	NS	NS	NI	NL					
19	Ductal	NS	NS	NI	LOH	5	25199	IVS5+15	A→G	Intronic
20	Ductal	NS	NS	CR	NL					
21	Ductal	IIA	2	CR	CR	5	25199	IVS5+15	A→G	Intronic
22	Ductal	NS	3	NI	NL					
23	Ductal	IIA	3	NI	NI	5	25199	IVS5+15	A→G	Intronic
24	Ductal	IIIB	2	NL	CR	5	25199	IVS5+15	A→G	Intronic
25	Ductal	IIA	2	NL	NL					

a Bases counted from ATG start codon; NS=not specified; NL=no loss; LOH=loss of heterozygosity; NI=not informative; CR=can't read; ND=not done.

). All patients were staged at surgery, in accordance with the recommendations of the American Joint Committee on Cancer (AJCC) and Union Internationale Contre le Cancer (UICC). The corresponding constitutional DNA was available in all cases from peripheral blood. Informed consent was obtained from all patients.

### DNA and RNA isolation

Cell lines were harvested for DNA and RNA extraction at about 80% confluence. Total RNA was extracted from breast and ovarian cell lines using the Tri-reagent (Sigma Castle Hill, New South Wales, Australia) following the manufacturer's instructions. PolyA+ RNA was prepared from total RNA using Dynabeads mRNA purification kit (Dynal, Carlton South, Victoria, Australia). For the primary ovarian tumours, tumour tissue was dissected free from necrotic and connective tissue and mechanically dispersed prior to collagenase treatment (0.1 mg ml^−1^ in Hanks balanced salt solution). Dead and red cells were then removed by Ficoll–Paque, and genomic DNA was extracted by the salting-out method (Miller *et al*, 1988). The purity of the resulting DNA is supported by the high frequency of LOH on chromosome 17 detected in ovarian tumour DNA prepared by this method (Leary *et al*, 1995). DNA was also extracted from peripheral blood and cell lines using the salting-out method. For breast tumours, DNA was extracted from snap-frozen pieces (containing at least 70% tumour cells) by proteinase K digestion, followed by phenol/chloroform extraction.

### Semiquantitative RT--PCR and Northern blot analysis

cDNA synthesis was primed with random hexamers and carried out on 1 *μ*g of total RNA using Superscript II (Promega). Primers pairs were designed to span at least one intron to avoid contamination from genomic DNA. RT–PCR was performed using 1 *μ*l cDNA incorporating ^33^P-dATP in a total volume of 20 *μ*l. Reactions were multiplexed with primers for *β-actin*, which served as an internal control. All primer sequences are listed in Table 1

**Table 1:** Primers used in the analysis of the BNIP3L gene

		**Sequence (5′–3′)**	**Size (bp)**
RT-PCR			
*BNIP3L*	F	ctgagtgccggagacggtcc	364
	R	ctgccatcttcttgtggcgaagg	
*β-actin*	F	cgtgacaataaggagaagctgtgc	375
	R	ctcaggaggagcaatgatcttgat	
Northern analysis			
*BNIP3L*	F	ctgagtgccggagacggtcc	993
	R	ctggcatttgcggaaaagaagccc	
*GAPDH*	F	atggatccagtccatgccatcactgcc	470
	R	atggtaccgaggtccaccaccctgttg	
Homozygous deletion analysis			
Exon 1	F	ctgactcgagcgtctccacgtccg	444
	R	cccatgcctgagccaatgagctgc	
Exon 2	F	gacagatttcagttctgctgtgg	295
	R	gtgaatgagccctgtaacccacctg	
Exon 3	F	gcagaacattttgggagtaagaatgc	462
	R	ccttagttgtaaaggagtgcg	
Exons 4 and 5	F	gagacacaaccttatgagtttgg	640
	R	caccacttcacaggtcacacgc	
Exon 6	F	gcggtacccacaaacctttagagcc	221
	R	caccacttcacaggtcacacgc	
DHPLC analysis			
Exon 1	F	ctgactcgagcgtctccacgtccg	444
	R	cccatgcctgagccaatgagctgc	
Exon 2	F	gacagatttcagttctgctgtgg	295
	R	gtgaatgagccctgtaacccacctg	
Exon 3	F	gcagaacattttgggagtaagaatgc	462
	R	ccttagttgtaaaggagtgcg	
Exon 4	F	gagacacaaccttatgagtttgg	259
	R	gcgtgcgcagatcaactgtgtcc	
Exon 5	F	cttgtcagtggacacagttgatctgc	414
	R	gtagaacaattgtgcaccc	
Exon 6	F	gcggtacccacaaacctttagagcc	221
	R	caccacttcacaggtcacacgc	

. Products were taken out at the end of cycles 20, 25 and 30 in order to assess amplification in the exponential phase, and these products were then run on a 5% denaturing acrylamide gel prior to autoradiography.

RNA was denatured and electrophoresed on a formaldehyde-agarose gel and transferred to a nylon membrane (Amersham Hybond N+) by capillary blotting overnight, then fixed to the membrane by ultraviolet irradiation according to standard protocols (Sambrook and Russell, 2001). Probe DNA was synthesised by RT--PCR (see Table 1 for primer details), and labelled with *α*^32^P-dCTP by random priming (Amersham, Megaprime kit, Castle Hill, New South Wales, Australia) before being hybridised to the membrane for 2 h in ExpressHyb solution (Clontech Inc., Palo Alto, CA, USA) at 65°C. The membranes were washed twice in 2×SSC/0.1% SDS at room temperature, then twice in 0.1×SSC/0.1% SDS at 65°C before autoradiography. Membranes were then stripped with 0.5% SDS and reprobed with GAPDH that served as an internal control.

Autoradiographs of RT–PCR and Northern blots were scanned at 300 dpi and the band intensity was determined by the ImageQuant program (Molecular Dynamics, Sunnyvale, CA, USA). Band intensity for *BNIP3L* was expressed as a proportion of the *β-actin* or *GAPDH* value for RT–PCR and Northern analysis, respectively, with the value for the reference epithelial cells (HOSE 17.1 or Bre-80-hTERT1) set to 1.0.

### Homozygous deletion analysis

Primers were designed to amplify the six exons of the *BNIP3L* gene (Table 1) and PCR performed on 20 ovarian and 15 breast cell lines. PCR products were visualised on an agarose gel. Samples were scored as deleted if a PCR failed when repeated with an internal control.

### LOH analysis

Analysis was carried out with the D8S137 and D8S1048 microsatellite markers that are located 1.4 and 0.55 Mb centromeric to the *BNIP3L* gene, respectively (http://www.celera.com). A measure of 5 ng of DNA was amplified by PCR for 35 cycles incorporating ^33^P-dATP. PCR products were run on a 5% denaturing acrylamide gel and then visualised by autoradiography. LOH was scored by two independent examiners as a reduction in the intensity of one allele by at least 50%. Any discrepancies between the two examiners were scored ‘can’t read' (CR).

### Denaturing high-performance liquid chromatography (DHPLC) analysis

Primers were designed to amplify the coding regions of all exons of the *BNIP3L* gene (Table 1). PCR products were amplified from 10 to 100 ng of genomic DNA using AmpliTaq Gold (PE Applied Biosystems) in a final volume of 20 *μ*l. Amplicons were then denatured at 95^o^C for 5 min and cooled to 60°C over 30 min (1°C min^−1^) prior to DHPLC. PCR products were loaded onto the autosampler and 5 *μ*l was injected onto the Varian Helix System (Varian, Walnut Creek, CA, USA). Samples were eluted within a linear acetonitrile gradient consisting of buffer A (0.1 M triethyl-ammonium acetate and 0.1 mM EDTA) and buffer B (0.1 M triethyl-ammonium acetate, 0.1 mM EDTA and 25% acetonitrile) with a flow rate of 0.45 ml min^−1^. The buffer B gradient was 45% (0–0.5 min), 50% (0.5–6 min), 68% (6–7 min), and 45% (7–8 min).

DHPLC was carried out at both the recommended melt tempe-rature as determined by the Stanford melt algorithm (http://insertion.stanford.edu/melt.html) and 2 above the recommended temperature. The recommended melt temperatures are 65°C for exon 1, 59°C for exon 2, 53 and 58°C for exon 3, 55 and 60°C for exon 4, 54 and 59°C for exon 5 and 60°C for exon 6. Analysis was performed using the Star Workstation version 5 (Varian, Walnut Creek, CA, USA). Samples that produced an aberrant shift in retention time and peak shape were repeated with their corresponding constitutional DNA to determine whether the shifts were tumour specific.

### Sequencing and cloning

All PCR products producing shifts on DHPLC were reamplified and sequenced with both forward and reverse primers using ABI Prism Big Dye Terminator cycle Sequencing Ready reaction kit (PE Applied Biosystem) and analysed on an ABI 377 sequencer. In some cases, PCR products were also cloned into the pGEM-T vector (Promega) prior to sequencing with the M13 forward (−21) and reverse primers.

## Results

### Characterisation of *BNIP3L* expression

**Figure 1:**
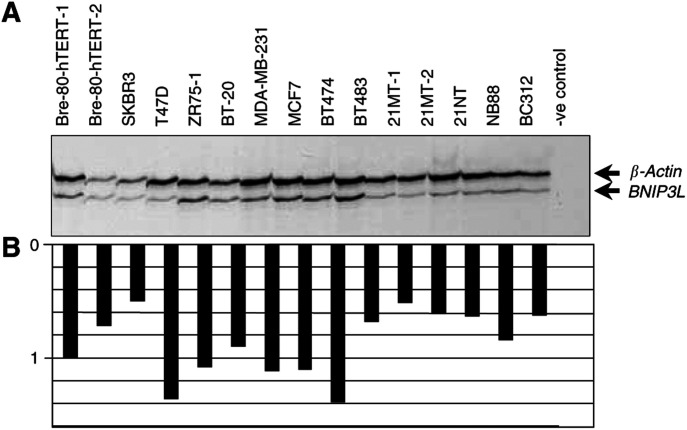
(**A**) Analysis of BNIP3L expression by RT–PCR in breast cancer cell lines. RT–PCR was carried out in a multiplex reaction with *β-actin* as an internal control for 20 cycles on cDNA from Bre-80-hTERT-1, Bre-80-hTERT-2 and 13 breast cancer cell lines. (**B**) Quantification of *BNIP3L* expression relative to *β-actin.*

*BNIP3L* expression was analysed by both RT–PCR and Northern blot analysis in breast and ovarian cancer cell lines, as well as in cell lines derived from the corresponding normal epithelial cells. RT–PCR was performed on the immortalised human breast epithelial cell lines Bre-80-hTERT1 and Bre-80-hTERT2 and 13 breast cancer cell lines (Figure 1A

). Expression was detected in both Bre-80-hTERT1 and Bre-80-hTERT2 and in all breast cancer cell lines at similar levels. Quantification of *BNIP3L* expression showed little variation in the 13 breast cancer cell lines when compared with two immortalised normal breast epithelial cell lines (Figure 1B).

**Figure 2:**
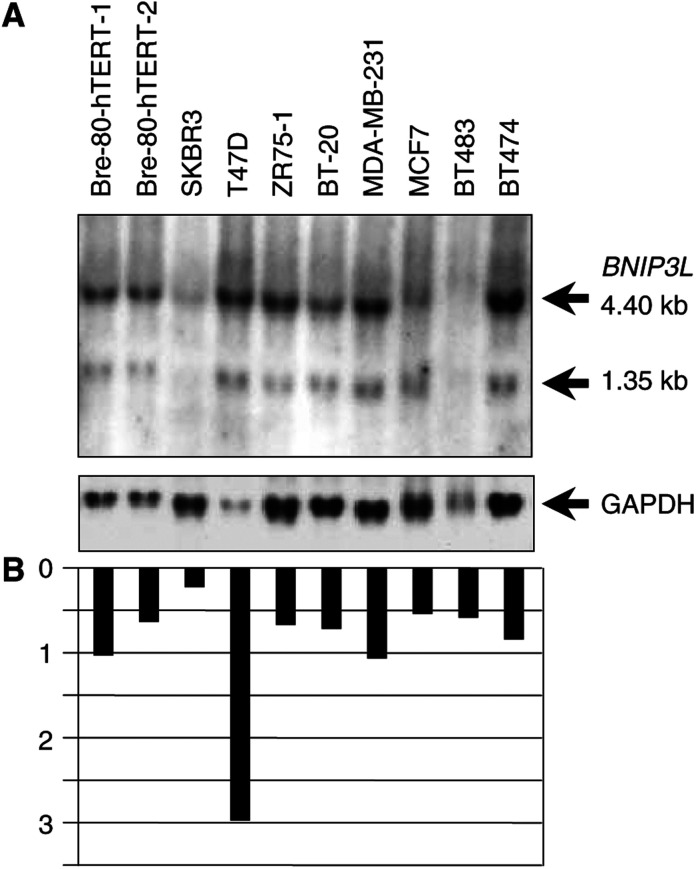
(**A**) Northern blot analysis of BNIP3L expression in breast cancer cell lines. Each lane represents 15 *μ*g of total RNA from Bre-80-hTERT-1, Bre-80-hTERT-2 and 8 breast cancer cell lines. (**B**) Quantification of *BNIP3L* expression relative to GAPDH.

*BNIP3L* expression was also examined by Northern blotting in a subset of eight of the breast cancer cell lines. Two transcripts of 1.3 and 4.4 kb were detected in the Bre-80-hTERT1 and Bre-80-hTERT2 cells and all breast cancer cell lines (Figure 2A

). Apart from T47D, in which expression was increased, there was little variation in the level of expression in the remaining breast cancer cell lines compared to the Bre-80-hTERT cells, although there was some variability in the relative intensity of each transcript (Figure 2B).

**Figure 3:**
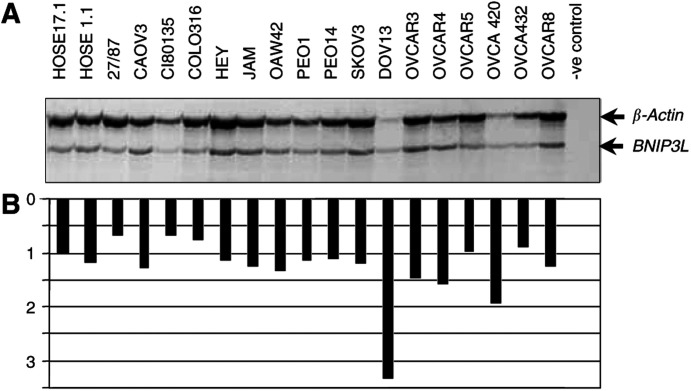
(**A**) Analysis of BNIP3L expression by RT–PCR in ovarian cancer cell lines. RT–PCR was carried out in a multiplex reaction with *β-actin* as an internal control for 20 cycles on cDNA from HOSE 17.1 and HOSE 1.1, and 16 ovarian cancer cell lines. (**B**) Quantification of *BNIP3L* expression relative to *β-actin.*

For the ovarian cancer analysis, RT–PCR was conducted on HOSE 1.1 and HOSE 17.1, and 17 ovarian cancer cell lines (Figure 3A

). Expression was detected in both HOSE cell lines and the 17 ovarian cancer cell lines. Of the 17 ovarian cancer cell lines, expression was notably different only in DOV-13, which showed about three-fold greater expression than the HOSE cell lines (Figure 3B).

**Figure 4:**
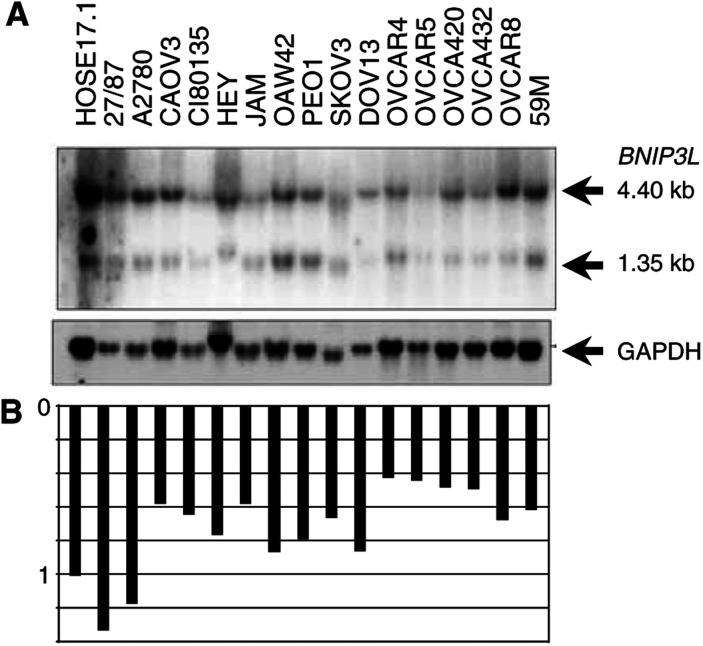
(**A**) Northern blot analysis of BNIP3L expression in ovarian cancer cell lines. Each lane represents 15 *μ*g of total RNA from HOSE 17.1 and 16 ovarian cancer cell lines. (**B**) Quantification of *BNIP3L* expression relative to GAPDH.

*BNIP3L* expression was also examined by Northern blot analysis in a subset of 16 ovarian cancer cell lines. As in the breast cell lines, two transcripts of 1.3 and 4.4 kb were detected in the HOSE 17.1 cell line and the 16 ovarian cancer cell lines (Figure 4A

). *BNIP3L* was expressed by all the ovarian cancer cell lines, but the level of expression was reduced to approximately half that of HOSE 17.1 in six of the 16 cancer cell lines (Figure 4B). No aberrant transcripts were detected by Northern blot analysis.

### Analysis of genetic alterations at the *BNIP3L* locus

No homozygous deletions within *BNIP3L* were found in any cancer cell line (data not shown). LOH analysis was carried out on 40 primary ovarian tumours and 25 primary breast tumours, and their corresponding constitutional DNA to assess allelic loss at the *BNIP3L* locus. Two microsatellite markers were used, D8S137 and D8S1048. LOH was observed for at least one of the markers in 14 out of 34 (41%) ovarian tumours. A frequency of 43% (10 out of 23) and 46% (12 out of 26) LOH was observed in informative ovarian tumours for D8S137 and D8S1048, respectively (Table 2). No correlation was found between LOH at either marker and tumour grade or histology. However, there was a statistically significant trend for LOH with later stage tumours (*P*=0.03). A frequency of 33% (four out of 12) and 15% LOH (two out of 13) was observed in informative breast tumours for D8S137 and D8S1048, respectively, with LOH observed for at least one marker in 28% (five out of 18) of the cases (Table 3). No correlation was found between LOH at either marker and tumour grade or stage.

### Mutation analysis

**Figure 5:**
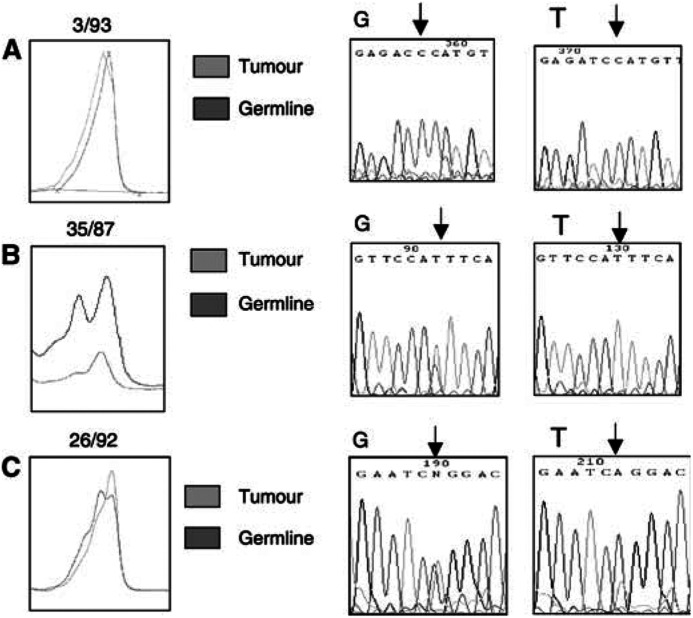
DHPLC shifts and sequencing results. (**A**) c.IVS2+11C>T somatic mutation in case 3/93. G=germline and T=tumour DNA. (**B**) c.407C>T polymorphism in case 35/87 showing LOH of the C allele. (**C**) c.181A>T polymorphism in case 26/92 showing LOH of the T allele.

Mutation analysis of *BNIP3L* was carried out on the same series of 40 primary ovarian tumours and 25 primary breast tumours by DHPLC. In the ovarian tumours, a total of five rare and one common polymorphisms were identified, as well as a single, intronic somatic mutation in case three out of 93. Direct sequencing of the PCR product from the three out of 93 tumour failed to show any change in nucleotide sequence, so the PCR product from both the tumour and constitutional DNA was cloned. A c.IVS2+11C>T conversion was detected in one out of four tumour clones and zero out of four constitutional DNA clones, suggesting that this mutation was somatic, which was consistent with the DHPLC chromatograph (Figure 5A

). Four of the polymorphisms were detected in coding regions in cases 35/87 (c.407C>T, Figure 5B), 26/92 (c.181A>T, Figure 5C), 12/93 (c.196C>A) and 61/93 (c.196C>G). These polymorphisms were all silent changes except for c.407C>T, which resulted in a change from leucine to phenylalanine. An intronic polymorphism involving a deletion and a substitution five bases apart was detected in case 35/90 (c.IVS3-56delG; c.IVS3-61T>C). A second, common, intronic polymorphism (IVS5+15T>C) was detected in 16/38 (42%) cases. In the breast tumours, only the common IVS5+15T>C polymorphism was detected in 13 out of 25 (52%) of the cases.

## Discussion

*BNIP3L* encodes a proapoptotic protein and is located at 8p21 (Matsushima *et al*, 1998; Chen *et al*, 1999; Imazu *et al*, 1999; Yasuda *et al*, 1999). *BNIP3L* reportedly induces cell death by altering mitochondrial membrane permeability and has been found to suppress clonicity in soft agar in cervical cancer cell lines (Matsushima *et al*, 1998). For these reasons, it is a good candidate for the 8p21 breast and ovarian cancer tumour suppressor gene.

RT–PCR and Northern blot analysis showed no evidence of frequent downregulation of *BNIP3L* expression in either breast or ovarian cancer cell lines. Northern blot analysis identified two transcripts of 1.3 and 4.4 kb, which were similar in size to those reported by Yasuda *et al*,(1999), who identified two transcripts of 1.6 and 3.9 kb. Other investigators have only observed one transcript of either 1.45 (Matsushima *et al*, 1998) or 4.5 kb (Chen *et al*, 1998). Yasuda *et al*,(1999) suggested that the two transcripts may be the result of alternative splicing or that one transcript may be derived from a closely related gene. We hypothesise that the larger transcript is an alternatively spliced form of the *BNIP3L* gene and hence quantitated *BNIP3L* expression as the sum of both transcripts.

Analysis of microsatellite markers near the *BNIP3L* locus detected frequencies of LOH of 43% (D8S137) and 46% (D8S1048) in the ovarian cancers. This is consistent with reported LOH frequencies of 45–58% at 8p21 in ovarian cancers (Lassus *et al*, 2001; Pribill *et al*, 2001). The frequency of LOH in the breast cancers of 27% (D8S137) and 28% overall is also consistent with a previous report of 30% LOH at D8S137 (Seitz *et al*, 2000), but lower than the 49% reported at nearby marker D8S1116 (Yokota *et al*, 1999). In some cases (35 out of 87 and 26 out of 92), the LOH scored at the microsatellite markers was confirmed by the DHPLC analysis and sequencing.

The frequency of LOH was significantly higher in late-stage ovarian tumours suggesting that LOH at the D8S137 and D8S1048 loci occur in the progression rather than the initiation of ovarian cancer. A higher incidence of LOH in larger breast tumours (50% LOH in tumours >5 cm in diameter *vs* 11% in tumours <5 cm in diameter) observed in a previous report (Seitz *et al*, 2000) was not observed in our study.

Mutation analysis was carried out by DHPLC, resulting in the identification of one somatic mutation and six polymorphisms in the ovarian tumours and one polymorphism in the breast tumours. The single somatic mutation, in exon two, is a silent mutation in the flanking intron and therefore is unlikely to affect the protein function. The majority of the polymorphisms (four out of six) occurred in the coding sequence, and of these, three were silent changes in the third base of a codon. The fourth was a nonconservative substitution of phenylalanine for leucine at codon 137. This variant was present in the constitutional DNA, but underwent LOH in the tumour. The remaining two polymorphisms occurred in introns and were not located in any donor or acceptor consensus sequences, and so are unlikely to affect splicing.

There are five polymorphisms currently listed for *BNIP3L* in the Genbank database (XM_048074), one of which occurs in the 5′ UTR, one in the coding sequence (codon 48, c288G>T), and three in the 3′ UTR. There are also two listed in the SNP consortium (http://snp.cshl.org/), one in the 5′ UTR and one in the first intron. Of these, only the polymorphism in the codon 48 could have been identified by our analysis and it was not detected in any of the cases analysed here.

The absence of mutations in *BNIP3L* in breast and ovarian tumours, and the lack of significant downregulation of the gene in either tumour type, suggests that *BNIP3L* is not the target of 8p LOH in ovarian and breast tumours, despite its location in the SROs of LOH in ovarian cancer (Brown *et al*, 1999) and breast cancer (Yokota *et al*, 1999; Seitz *et al*, 2000). A number of other candidate tumour suppressor genes are also located within this region (*STC1*, *ADAMDEC1*, *EXTL3*, *DOK2*, *DPYSL2*, *CLU* and *NKX3A*), but further refinement of the region by LOH analysis of primary tumours or monochromosome-mediated chromosome transfer (MMCT) may be necessary before identification of the tumour suppressor is possible.
